# Intelligent Design of Building Materials: Development of an AI-Based Method for Cement-Slag Concrete Design

**DOI:** 10.3390/ma15113833

**Published:** 2022-05-27

**Authors:** Fei Zhu, Xiangping Wu, Mengmeng Zhou, Mohanad Muayad Sabri Sabri, Jiandong Huang

**Affiliations:** 1 Department of Gem Design Engineering, KAYA University, Gimhae 50830, Korea; zhufeiedu@163.com (F.Z.); xiangping_wu@163.com (X.W.); 2 Xuzhou Finance and Economics Branch, Jiangsu Union Technical Institute, Xuzhou 221116, China; 3 School of Materials Engineering, Xuzhou College of Industrial Technology, Xuzhou 221116, China; 4 School of Mines, China University of Mining and Technology, Xuzhou 221116, China; ts21020232p21@cumt.edu.cn; 5 Peter the Great St. Petersburg Polytechnic University, 195251 St. Petersburg, Russia; mohanad.m.sabri@gmail.com

**Keywords:** beetle antennae search, multi-objective optimization, random forest, decision tree, uniaxial compressive strength

## Abstract

Cement-slag concrete has become one of the most widely used building materials considering its economical advantage and satisfying uniaxial compressive strength (UCS). In this study, an AI-based method for cement-slag concrete design was developed based on the balance of economic and mechanical properties. Firstly, the hyperparameters of random forest (RF), decision tree (DT), and support vector machine (SVM) were tuned by the beetle antennae search algorithm (BAS). The results of the model evaluation showed the RF with the best prediction effect on the UCS of concrete was selected as the objective function of UCS optimization. Afterward, the objective function of concrete cost optimization was established according to the linear relationship between concrete cost and each mixture. The obtained results showed that the weighted method can be used to construct the multi-objective optimization function of UCS and cost for cement-slag concrete, which is solved by the multi-objective beetle antennae search (MOBAS) algorithm. An optimal concrete mixture ratio can be obtained by Technique for Order Preference by Similarity to Ideal Solution. Considering the current global environment trend of “Net Carbon Zero”, the multi-objective optimization design should be proposed based on the objectives of economy-carbon emission-mechanical properties for future studies.

## 1. Introduction

Concrete is widely used in modern construction projects because of its low price, simple production process, high UCS, good durability, and other characteristics [1]. Although concrete is benefiting humankind, it also brings some negative effects to the environment [2]. Cement is the main cementitious material of concrete and a lot of energy and resources are consumed in the process of cement production [3,4]. The large amount of carbon dioxide emitted in the process of cement production is a serious burden and threat to the environment [5,6,7]. Finding new materials to replace some of the cement used in concrete is a necessary way to ensure the sustainable development of the concrete industry [8,9,10,11,12,13,14,15,16]. At present, the use of fly ash, blast furnace slag, metakaolin, and other mineral admixtures to replace part of the cement used in concrete is the main solution to alleviate the large resource consumption and negative impact on the environment in the cement production process [17,18,19,20,21,22]. Blast furnace slag is the most common mineral admixture and a type of industrial waste slag discharged from the blast furnace when smelting pig iron, so it has the characteristics of low environmental damage and low price [23,24,25]. The concrete mixed with blast furnace slag has reduces the amount of cement required, improves the performance of concrete, reduces the cost of concrete, and reduces the damage of industrial waste slag on the environment. Therefore, it is of great significance to study concrete mixed with blast furnace slag [26]. 

Typically, engineers use the laboratory test method to design high-performance concrete to meet the mechanical characteristics of high UCS, large flow performance, good durability, and so on [27,28]. However, the laboratory test method is of high cost, time consuming, and effort consuming [29]. In particular, when multiple properties of concrete need to be optimized, the number of samples that need to be designed will increase exponentially, and the shortcomings of laboratory experimental methods will be more prominent [30,31,32,33]. The UCS of concrete refers to the maximum pressure per unit area of the standard specimen that can bear pressure until it is destroyed [34,35]. It is one of the most important properties of concrete. The relationship between the UCS of concrete and the proportion of the admixture is non-linear, so it cannot be simply calculated by a mathematical formula [36,37]. Many researchers have proposed the use of machine learning to achieve more efficient optimization of the UCS of concrete [10,38,39,40,41,42,43,44,45,46,47,48,49,50]. Zhang et al. proposed a new self-organizing fuzzy neural network (SOFNN) method based on clustering and extreme learning machine (ELM) optimization to overcome the fact that traditional machine learning models are difficult for engineers to understand when predicting the UCS of concrete. Kumar et al. studied the use of Gaussian progress regression (GPR), support vector machine regression (SVMR), and ensemble learning (EL) to predict the UCS of concrete. The research results showed that the optimized GPR model has great accuracy in predicting the UCS of concrete, and the optimized SVMR and GPR models also show good performance in predicting the UCS of concrete [51]. Song et al. proposed the use of random forest (RF) and gradient-enhanced regression (GBR) to predict the UCS of concrete containing fly ash, and the research results showed that the models have a good effect on predicting the UCS of concrete containing fly ash [52]. Silva et al. proposed three models—random forest (RF), support vector machine (SVM), and artificial neural network (ANN)—to predict the UCS of concrete, and compared the results with other models. The results showed that the three models are better [29]. The above research results showed that the machine learning method has a good effect on predicting the UCS of concrete. However, it should be noted that the above studies only focused on the mechanical properties of concrete and that other design objectives (such as economy or carbon emission) were not considered synergistically.

Although the price of concrete is low, with the increasing use of concrete in the construction industry, the cost of concrete is also a significant expense for the construction industry. There is a linear relationship between concrete price and mix ratio, so the optimization of concrete cost can be studied by the formula. However, there are few studies on cost optimization of concrete, and even fewer studies on multi-objective optimization of UCS and cost. To solve the above problems, this paper proposes a multi-objective optimization the beetle antennae search (MOBAS) algorithm to optimize the UCS and cost of the concrete mixture [53]. For the optimization of the UCS of concrete, BAS is firstly used to tune the hyperparameters of SVM, RF, and DT, and then the model with the best prediction effect of concrete UCS is found from the above three models. The white box is opposite to the black box problem used for artificial intelligence prediction. It refers to a mathematical problem with a definite correspondence between input variables and output variables, which can be solved forward. Using the input parameters from the raw materials, the output parameter (cost) can be obtained directly by the white box.

**Figure 1:**
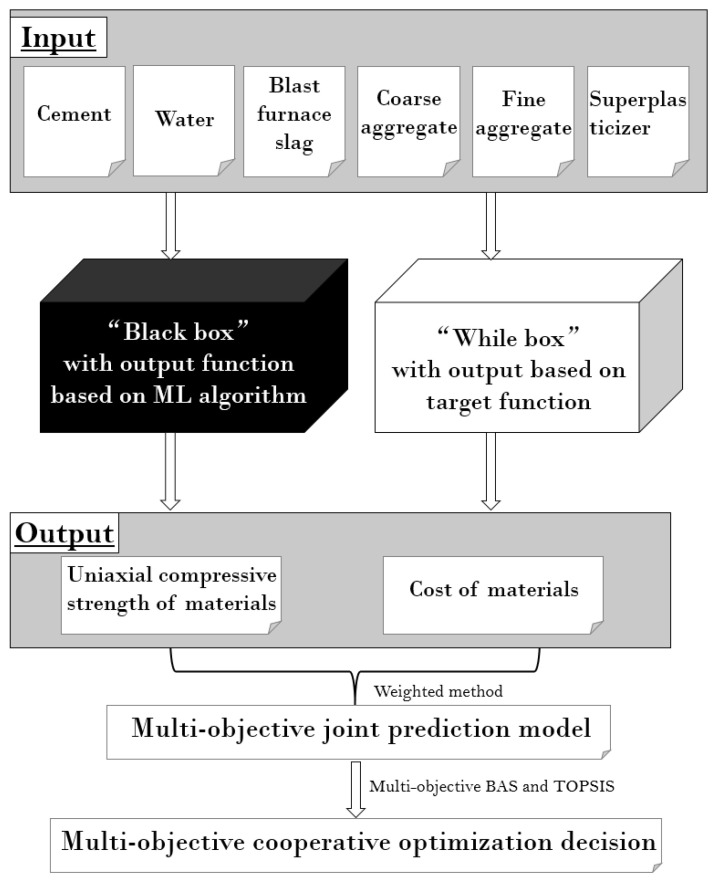
Framework diagram of the multi-objective optimization model.

For the optimization of the cost of concrete, the decision variables, objective functions, and constraint conditions need to be defined first, and then the model is built according to the relationship between cost and admixture. There is a big difference between single-objective optimization and multi-objective optimization. The non-dominant solution that is suitable for single-objective optimization can be applied to multi-objective optimization only under special conditions. Multi-objective optimization usually requires high computing power to obtain the non-dominant solution set and the Pareto frontier. Additionally, the optimization of the concrete mixture will be affected by the curing environment, performance, material type, and so on. To solve the above problems, this study proposed the use of a weighted method to construct the MOBAS algorithm to solve the optimal solution of the multi-objective function value. Finally, Technique for Order Preference by Similarity to Ideal Solution (TOPSIS) was used to determine the final optimal mix ratio of the concrete optimization problem. The framework of the multi-objective optimization model in this study is shown in Figure 1.

## 2. Research Methods

### 2.1. Description of the Dataset

**Figure 2:**
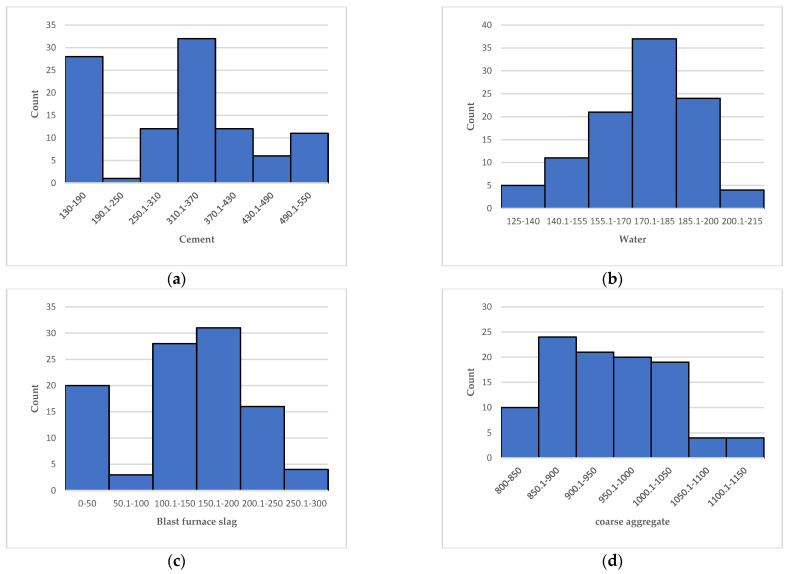

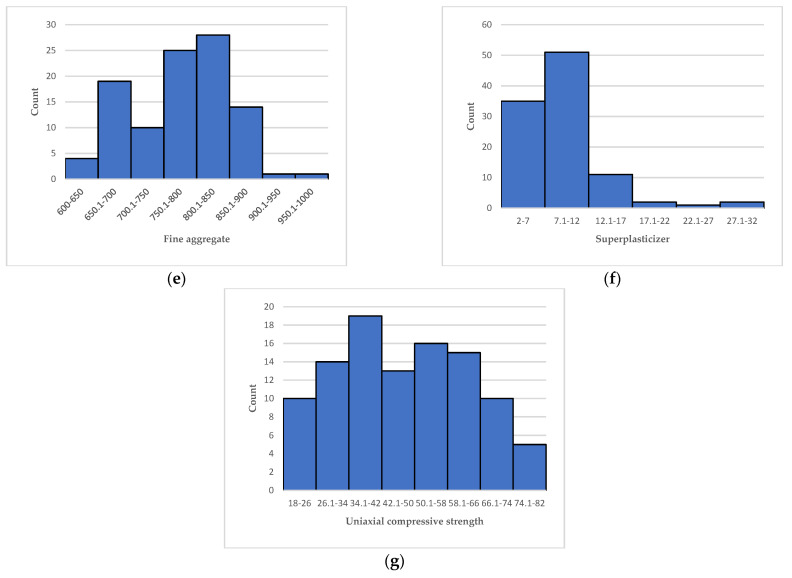
Frequency distribution histogram of variables. (**a**) Cement; (**b**) Water; (**c**) Blast furnace slag; (**d**) Coarse aggregate; (**e**) Fine aggregate; (**f**) Superplasticizer; (**g**) UCS.

A reliable database is a basis for verifying the accuracy of prediction models for the UCS of concrete. This study collected 102 datasets from previously published articles to form the database [54]. In this database, cement, water, blast furnace slag, coarse aggregate, fine aggregate, and superplasticizer are the input variables, and UCS is the output variable [55,56]. The distribution of data in the dataset is also a key factor to determine whether the database is reliable. To achieve a more intuitive understanding of the distribution of data in the database, this study presents the data analysis table and the frequency distribution histogram of the seven variables in Table 1 and Figure 2, respectively. Table 1 shows that the average content of cement, water, blast furnace slag, coarse aggregate, fine aggregate, superplasticizer and UCS in the database is 311.65, 174.43, 117, 946.50, 744.03.95, 9.13 and 47.67 kg/m^3^, respectively. From Figure 2, it can be seen more intuitively that the data distribution of the seven variables covers a wide range and is reasonable. Therefore, using this database to predict the UCS of concrete can achieve good results.

### 2.2. Algorithm

#### 2.2.1. The Beetle Antennae Search Algorithm (BAS)

The BAS algorithm is a new meta-heuristic algorithm that is inspired by beetle search behavior during foraging. The long antennae of beetles usually contain several odor-picking cells that can be used to detect the scent of prey. Beetles wiggle their antennae to locate food while foraging. If the intensity of the smell received by the left tentacle is greater than that received by the right, the beetle will fly to the left, otherwise, it will fly to the right to find food.

**Figure 3:**
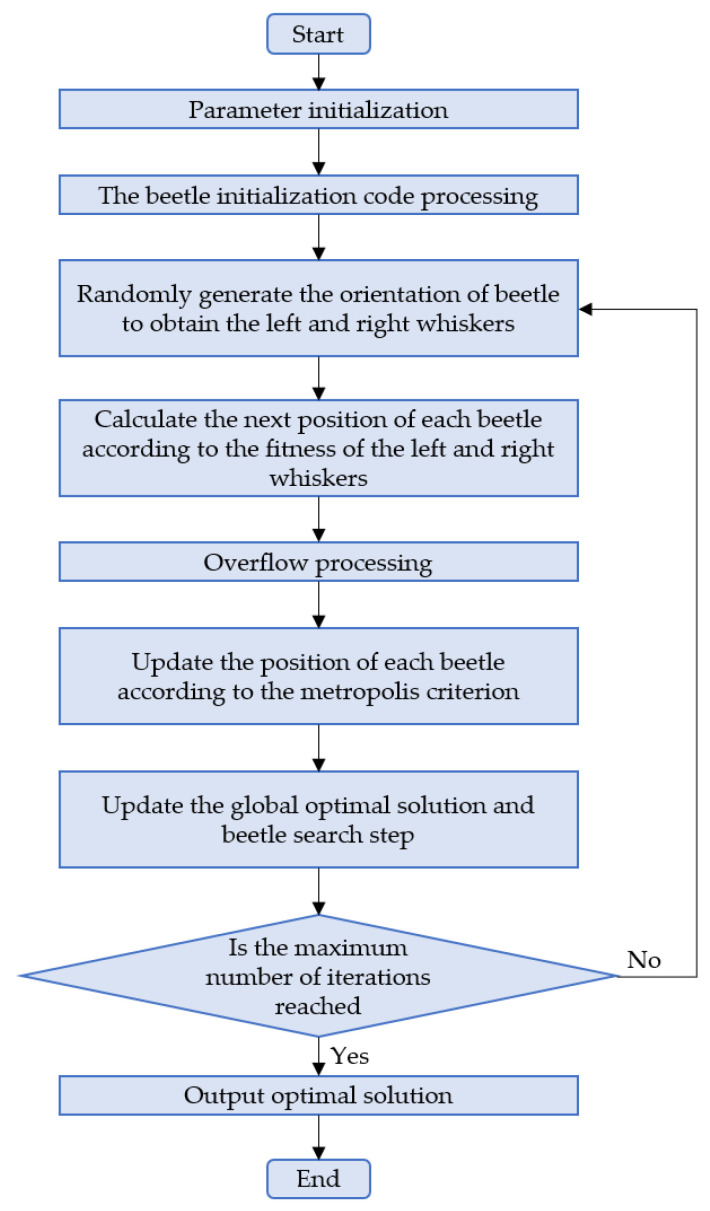
The flow chart of BAS.

In the BAS algorithm, the objective function to be optimized is regarded as food, and the variable of the objective function is regarded as the location of the beetles. For unknown areas, the beetles usually use random search to generate random directions. This process is stimulated by the following formula:

(1) $$\vec{b}=\frac{rnd(d,1)}{\left\|rnd(d,1)\right\|}$$

where rnd(⋅) is a random function, and the number produced is between −1 and 1; *d* is the dimension of the variable. The formula for calculating the spatial coordinates of the left and right antennae of beetles is as follows:

(2) $$\left\{ \begin{array}{l} x_{l}^{t}=x^{t}+h^{t}\cdot \vec{b}\\ x_{r}^{t}=x^{t}-h^{t}\cdot \vec{b} \end{array}\right.$$

where $x_{l}^{t}$ and $x_{r}^{t}$ represent the position of the left whisker and right whisker of the beetle at time t, respectively, and $x_{t}$ is the position of the beetle at time *t*. The position of the beetle is as follows:

(3) $$x^{t+1}=x_{t}-\delta^{t}\cdot \vec{b}\cdot sign(f(x_{l}^{t})-f(x_{r}^{t}))$$

where f(⋅) is the fitness function, and $f(x_{l}^{t})$ and $f(x_{r}^{t})$ are the odor concentration at the left and right whiskers of the beetle, respectively. $\delta^{t}$ is the step size at time *t*, and sign(⋅) is the sign function. The beetle whisker length *h* and step size δ decrease with the increase in iteration number. A possible new approach is as follows:

(4) $$h^{t}=r_{h}\cdot h^{t-1}+0.01$$

(5) $$\delta^{t}=r_{\delta }\cdot \delta^{t-1}$$

where the initial values of *h* and δ, and their corresponding attenuation coefficients $r_{h}$ and $r_{\delta }$, need to be selected according to the range of functions to be optimized. The flow chart of the BAS algorithm is shown in Figure 3.

#### 2.2.2. Random Forests (RF)

The RF model is a typical ensemble learning algorithm. It is widely used in various fields because of its advantages in being easy to understand, ease of application, and lack of over-fitting. The construction process of the RF algorithm: assume that the training dataset $D=\left\{\left(x_{1},y_{1}),(x_{2},y_{2}),\cdots ,(x_{n},y_{n}\right)\right\}$ is known and the sample feature number is *m*. The bootstrap sampling method is used to extract K datasets with size n from the training dataset *D*, and train K decision tree models, respectively. In the process of spanning the tree, mtry ($mtry=\log_{2}m$ or $\sqrt{m}$) features are usually selected each time when dividing nodes. The classification of feature nodes needs to select the most important features according to the feature evaluation method.

#### 2.2.3. Decision Tree (DT)

DT is a typical classification method. In essence, the method classifies data through a series of rules. The method first needs to process the data, then use the inductive algorithm to generate readable rules and decision trees, and use the generated decision tree to analyze the new data. DT mines rule in data by constructing the decision tree and then classifying data. The construction of the decision tree consists of two steps: the generation of the decision tree and the pruning of the decision tree. The generation of the decision tree is the process of generating a decision tree from training dataset. Decision tree pruning refers to the process of verifying and correcting the decision tree generated in the previous step with the test dataset.

#### 2.2.4. Support Vector Machine (SVM)

SVM is an efficient classification model, which constructs the classification hyperplane through learning training data in the case of linear separable. The classification hyperplane is defined as:

(6) $$f(t)=\omega^{t}x+b$$

Then, the new data point $x^{\prime }$ is classified. If $f(x^{\prime })>0$, then $x^{\prime }$ is a positive case; if $f(x^{\prime })<0$, then $x^{\prime }$ is a negative case. Suppose the training dataset $T=\left\{\left(x_{1},y_{1}),(x_{2},y_{2}),\cdots ,(x_{m},y_{m}\right)\right\}$, in which $x_{i}\in \delta^{n}$, $y_{i}\in \left\{-1,1\right\},i=1,2,\cdots m$, *m* represents the number of data points. The classification hyperplane solution formula of hard interval support vector machine is as follows:

(7) $$\underset{\omega ,b}{\min}=\frac{1}{2}{\left\|\omega \right\|}^{2}$$

(8) $$s.t.y_{i}(\omega^{T}x_{i}+b)\geq 1,i=1,\cdots m.$$

However, there are usually some outliers in the training set. To solve these outliers, it is necessary to further improve the robustness of the model, and then a soft interval support vector machine model is proposed. The solution formula is as follows:

(9) $$\underset{\omega ,b,\xi_{i}}{\min}\frac{1}{2}{\left\|\omega \right\|}^{2}+C\sum_{i=1}^{m}\xi_{i}$$

(10) $$s.t.y_{i}(\omega^{T}x_{i}+b)\geq 1-\xi_{i}$$

(11) $$\xi_{i}\geq 0,1=1,2,\cdots ,m.$$

where $\xi_{i}$ is the relaxation variable of the ith training point, *C* is the regularization parameter, and C>0.

### 2.3. Construction of the Cost Objective Function

The cost of concrete is mainly modeled and optimized through mathematical formulas. It is calculated as follows:

(12) $$COST=C_{C}Q_{C}+C_{w}Q_{W}+C_{SF}Q_{SF}+C_{CA}Q_{CA}+C_{FA}Q_{FA}+C_{sp}Q_{sp}$$

where $C_{C},C_{w},C_{SF},C_{CA},C_{FA},C_{SP}$ are the unit price of cement, water, blast furnace slag powder, coarse aggregate, fine aggregate, and superplasticizer, respectively, and $Q_{C},Q_{W},Q_{SF},Q_{CA},Q_{FA},Q_{SP}$ are the amount of cement, water, blast furnace slag powder, coarse aggregate, fine aggregate, and superplasticizer per cubic meter of concrete, respectively. The cost and densities of concrete materials are shown in Table 2.

To solve the multi-objective optimization problem, after determining the objective function of concrete cost optimization, the next step is to determine the constraint conditions. The constraints are as follows:

(1) Scope constraints

In the optimization process of the concrete mix ratio, the decision variables need to be constrained according to the range of data in the database, namely:

(13) $$D_{i\min}\leq D_{i}\leq D_{i\max}$$

where $D_{i}$ is the ith decision variable, $D_{i\min}$ and $D_{i\max}$ are the minimum and maximum values of the ith decision variable in the database used in this study, respectively.

(2) Proportional constraints

In the optimization process of the concrete mixture problem, several proportions need to be restricted according to the data of the database used in this study including the proportion of water to cementitious material (W/C + SF), the proportion of blast furnace slag to cementitious material (SF/C + SF), the proportion of superplasticizer to cementitious material (SP/C+SF), the proportion of coarse aggregate to aggregate (CA/CA + FA), and the proportion of coarse aggregate to cementitious material (CA/C + SF). The range of proportion relation of each component is shown in Table 3.

(3) Volume constraint

Taking the sum of the total volume of each component in concrete as equal to one, set the concrete volume constraint equation as follows:

(14) $$V_{m}=\frac{Q_{C}}{U_{C}}+\frac{Q_{W}}{U_{W}}+\frac{Q_{SF}}{U_{SF}}+\frac{Q_{CA}}{U_{CA}}+\frac{Q_{FA}}{U_{FA}}+\frac{Q_{SP}}{U_{SP}}$$

where $U_{C},U_{W},U_{SF},U_{CA},U_{FA},U_{SP}$ are the unit weight of cement, water, blast furnace slag powder, coarse aggregate, fine aggregate, and superplasticizer, respectively.

(4) UCS constraint

In this optimization problem, the UCS of concrete must be greater than the design value, so the UCS of concrete is constrained as follows:

(15) $$f_{cg}\leq f_{c}\leq f_{cg}^{\prime }$$

where $f_{c}$ is the predicted UCS of concrete, $f_{cg}$ is the given UCS of concrete, and $f_{cg}^{\prime }$ is the upper limit of UCS of concrete.

### 2.4. The Multi-Objective BAS Algorithm

#### 2.4.1. Multi-Objective Problem

The multi-objective optimization problem is a field of multi-criteria decision making, which refers to the simultaneous optimization of multiple objective functions. Its mathematical expression is as follows:

(16) $$\left\{ \begin{array}{l} f\left(X\right)=\left(f_{1}(x),f_{2}(x),\ldots ,f_{M}(x)\right)\\ g_{i}(x)=0\\ h_{j}(x)\geq 0 \end{array}\right.$$

where *X* is the decision variable, $f_{1}(x),f_{2}(x),\cdots ,f_{m}(x)$ is the objective function to be optimized, which may conflict with each other, $g_{i}(x)$ is the equality constraint to be satisfied, and $h_{j}(x)$ is the inequality constraint to be satisfied.

Because the multi-objective optimization problem needs to optimize multiple objective attributes at the same time, it cannot be judged by comparing the size relationship between two individuals like the single-objective optimization problem. Therefore, dominant relations are defined to judge the merits of different goals. In the minimization multi-objective optimization problem, suppose individuals $x_{1}$ and $x_{2}$, exists for all sub-targets; in other words, all the sub-target function values of $x_{1}$ are less than or equal to that of $x_{2}$, and there is at least one sub-target that the corresponding function value of $x_{1}$ is less than that of $x_{2}$, then $x_{1}$ dominates $x_{2}$.

#### 2.4.2. Pareto Optimality

**Figure 4:**
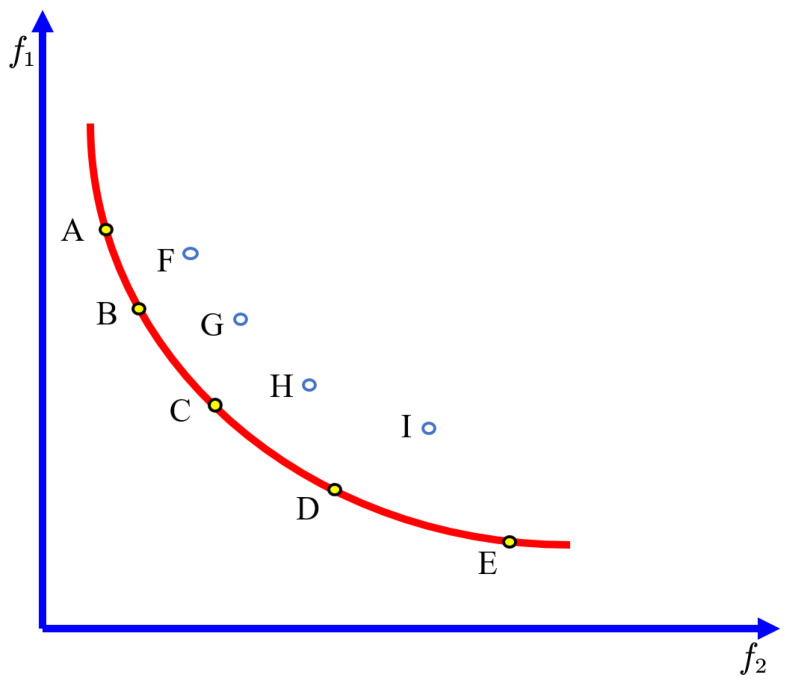
The Pareto frontier demonstration.

In multi-objective optimization problems, there are conflicting objectives, that is, if the sub-objective of one non-dominant solution is improved, the optimization effect of other sub-objective function values will be reduced. Therefore, the optimal solution obtained by a multi-objective optimization problem is usually not unique, but the optimal solution set is composed of multiple non-dominant solutions, also called Pareto optimal solution set. The solutions in the Pareto solution set are not dominated by other solutions, and relative to other solutions, at least one objective is optimal. The distribution of the Pareto solution, dominant solution, and Pareto solution boundary in the case of two objectives is shown in Figure 4.

Shown in Figure 4A–E are Pareto solutions of objective functions $f_{1}$ and $f_{2}$, and they are not dominated by other solutions, that is, they are non-dominated solutions.

#### 2.4.3. The Multi-Objective BAS Algorithm

**Figure 5:**
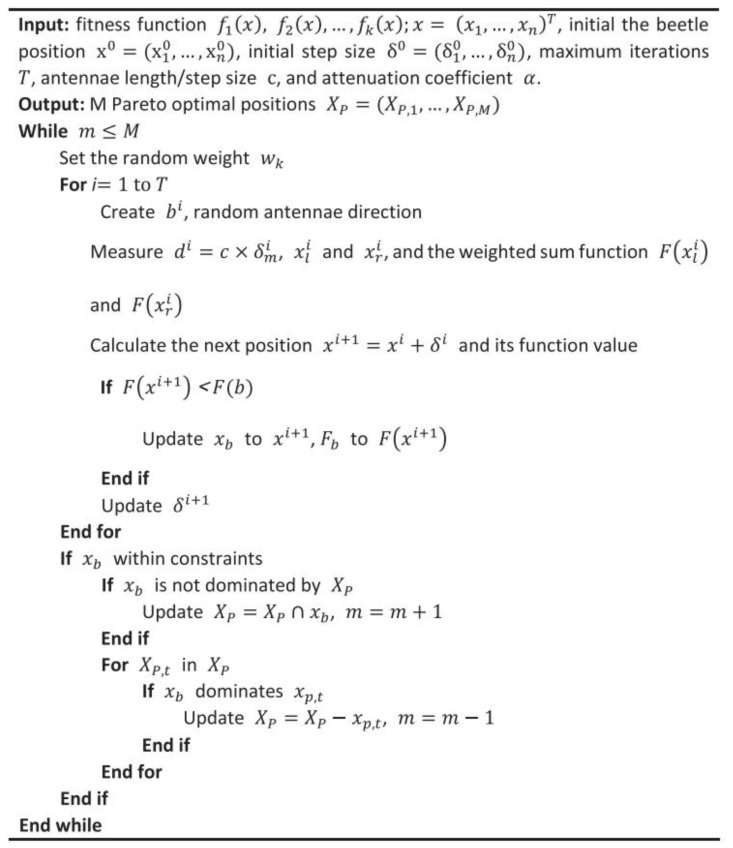
Multi-objective BAS algorithm [57].

In this study, the weighting method is used to combine multiple targets into single targets, and the formula used is as follows:

(17) $$f=\sum_{k=1}^{K}\omega_{k}f_{k},\sum_{k=1}^{K}\omega_{k}=1$$

where $\omega_{k}$ is the weight of the *k*th objective function, which is randomly generated from the uniform distribution, and $\omega_{k}\in \left[0,1\right]$. To achieve multi-objective BAS, *n* beetle populations are initialized first, and then target values of beetles are compared. The best solution can be obtained by generating random weights. After the iteration, *n* non-dominant solution points can be used to approximate the real Pareto front. Figure 5 presents the multi-objective BAS algorithm.

#### 2.4.4. Decision-Making Method

TOPSIS is the most commonly used multi-criteria decision analysis method in multi-objective decision analysis. TOPSIS is a method for evaluating relative merits and demerits among a limited number of objects. This method sorts the finite number of evaluation objects according to their proximity to the ideal target. By comparing the alternatives with the positive ideal solution (the optimal solution) and the negative ideal solution (the worst solution), the optimal solution closest to the positive ideal solution and furthest from the negative ideal solution is found. Additionally, rank the different solutions according to the proximity coefficient. The positive ideal point ($d_{i+}$), the negative ideal point ($d_{i-}$), and the proximity coefficient ($C_{i}$) can be calculated as follows:

(18) $$d_{i+}=\sqrt{\sum_{j=1}^{n}{\left(F_{ij}-F_{j}^{ideal}\right)}^{2}}$$

(19) $$d_{i-}=\sqrt{\sum_{j=1}^{n}{\left(F_{ij}-F_{j}^{non-ideal}\right)}^{2}}$$

(20) $$C_{i}=\frac{d_{i-}}{d_{i+}+d_{i-}}$$

where *i* represents a Pareto solution, *n* represents the number of objectives, $F_{j}^{ideal}$ represents the *j*th ideal solution in the single-objective optimization problem, and $F_{j}^{non-ideal}$ represents the non-ideal solution in the single-objective optimization problem. The solutions are sorted according to the size of $C_{j}$. The larger the $C_{j}$, the better the corresponding solution.

## 3. Results and Analysis

### 3.1. Hyperparameter Tuning

**Figure 6:**
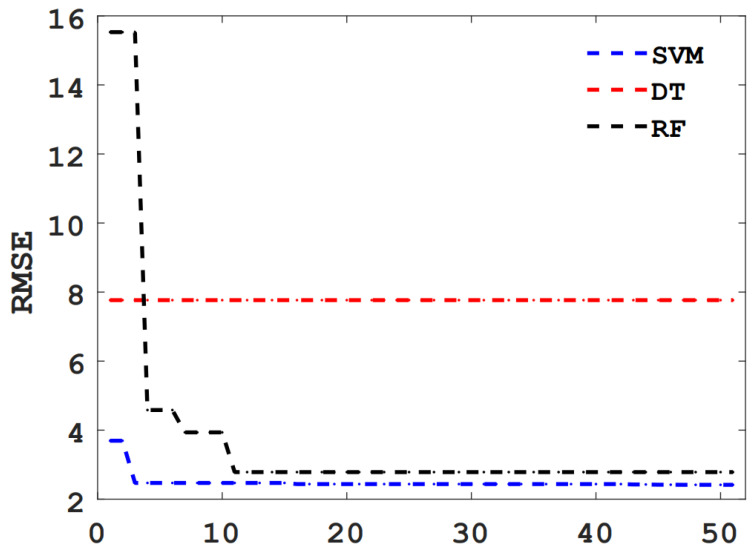
RMSE values for different ML models.

In this study, BAS is used to tune the hyperparameters of the RF, DT, and SVM models, and the relationship between iteration times and RSME values is shown in Figure 6. As can be seen from Figure 6, with the increase in the number of iterations, the RSME values of the DT model do not change at all, while the RSME values of RF and SVM drop sharply at first and tends to be stable after falling to a low value, and the RMSE value of SVM model is lower. The results show that BAS has a poor effect on DT hyperparameter tuning, but a better effect on SVM and RF hyperparameter tuning.

### 3.2. Evaluation of UCS Prediction Model

**Figure 7:**
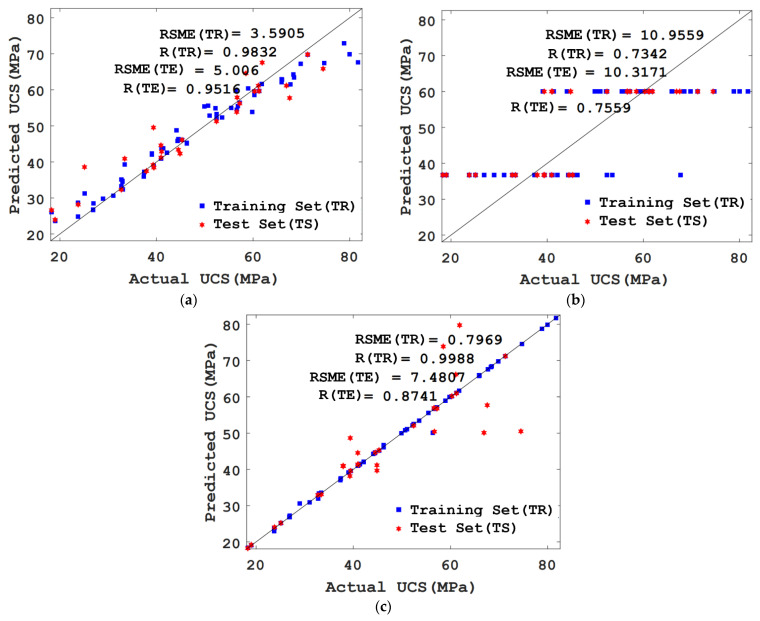
Comparison of prediction effects of different models on UCS of concrete. (**a**) RF model; (**b**) DT model; (**c**) SVM model.

In this study, the model with the best prediction effect was selected by comparing the predicted value and actual value of the RF, DT, and SVM models for the UCS of concrete. Figure 7 shows the comparison results between the predicted value and actual value of the RF, DT, and SVM models for the UCS of concrete. As shown in the figure, RF and SVM have a good consistency between the predicted value and actual value of the UCS of concrete, while DT has a poor consistency between the predicted value and actual value of the UCS of concrete. To select the model with a better prediction effect on the UCS of concrete from RF and SVM, this study further compared the R values and RSME values of the training set and test set of the two models. The R values of the training set and test set of the RF model are 0.9832 and 0.9516, and RSME values are 3.5905 and 5.006, respectively. The R values of the SVM training set and test set are 0.9988 and 0.8741, and RSME values are 0.7969 and 7.4807, respectively. Although SVM in the training set has a higher R value and a lower RSME value than RF, the R value of SVM in the test set is lower than the RF model, and the RMSE value of SVM is higher than the RF model. In addition, the R value of the test set in the SVM model is significantly lower than that of the training set, and the RSME value is significantly higher, which means that the UCS of concrete predicted by SVM appears over-fitting phenomenon.

**Figure 8:**
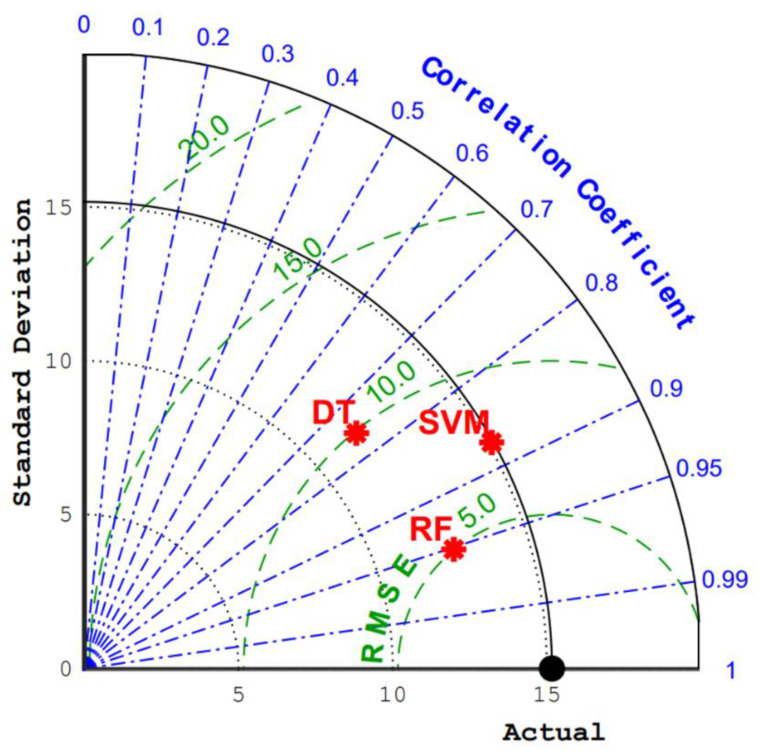
Taylor diagrams of test sets for different models.

Figure 8 more intuitively shows the comparison of R values and RSME values of RF, DT, and SVM in the test set. It can be seen that RF has a higher R value and a lower RSME value in the test set. It is proved that RF has a better prediction effect on the UCS of concrete. After a comparison of the RF, DT, and SVM models, the RF model was selected in this study to predict the UCS of concrete.

### 3.3. Evaluation of Multi-Objective Optimization Models

**Figure 9:**
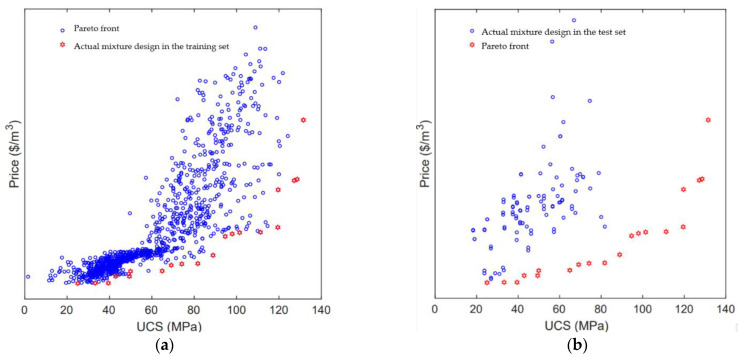
Pareto front of the concrete mixtures. (**a**) Training set; (**b**) Test set.

The optimization results of the UCS and the cost of concrete are shown in Figure 9. It can be seen that the Pareto frontier optimal solutions of the UCS and cost of concrete and the UCS and cost of concrete correspond to the actual data in the training set and the test set, respectively. The actual data of concrete in the training concentration are widely distributed and within a reasonable range. The actual points in the training set are located above the Pareto front optimal solution, which proves that the cost of reaching the specific UCS at the actual points in the training set is higher than the cost of reaching the UCS in the Pareto front optimal solution. Similarly, the actual UCS and cost data of concrete in the test dataset are not as wide as the training dataset, but the overall distribution is also reasonable. The cost of the actual data in the test set to reach a specific UCS is also higher than that of the Pareto frontier optimal solution to reach the UCS. The above results prove that the multi-objective optimization model proposed in this paper can effectively reduce the cost of concrete without changing the UCS of concrete, that is, the multi-objective optimization model has a good effect and strong generalization ability.

Since the UCS and cost of concrete are two conflicting goals, with the increase in cement content in concrete, the UCS of concrete also increases. However, due to the relatively high cost of cement, the cost of concrete also increases. To find the best mixture ratio for the optimization of the UCS and cost of concrete, this paper uses TOPSIS to rank the scores of twenty Pareto front optimal solutions. The TOPSIS scores of each point of Pareto front optimal solutions are shown in Table 4. It can be seen that the score of the Pareto front optimal solution with the highest score is 1. At this time, the UCS of the corresponding concrete is 119.48 MPa and the cost is 37.29 USD/m^3^. The contents of cement, water, blast furnace slag, coarse aggregate, fine aggregate and superplasticizer in the optimal concrete mixture are 484.21, 191.44 kg/m^3^, 239.70, 1080.26 kg/m^3^, 983.94 kg/m^3^, and 3.72 kg/m^3^, respectively.

## 4. Conclusions

It is a challenge in civil engineering to improve the performance of concrete by designing the proportion of the concrete mixture. As one of the most widely used building materials, economical advantage and satisfactory UCS were considered in the present study for the concrete design. An AI-based method for cement-slag concrete design based on the balance of economic and mechanical properties was developed and a multi-objective optimization model was proposed to optimize the proportion design of the concrete mixture. Based on the developed machine learning process, the following conclusions can be drawn:

(1) BAS is used to tune the hyperparameters of RF, DT, and SVM. The RSME values of RF and SVM converge gradually with the increase in the number of iterations, while the RMSE value of DT does not change with the increase in the number of iterations, demonstrating that BAS has a poor effect on DT hyperparameter tuning, but a better effect on SVM and RF hyperparameter tuning.
(2) By comparing the predicted value and actual value of RF, DT, and SVM optimized by BAS, it is found that RF and SVM have a better prediction effect on concrete UCS. However, due to the over-fitting of SVM, the RF model was selected in this study to predict the UCS of concrete considering the R values of the training set and test set of the RF model are 0.9832 and 0.9516, respectively.
(3) To find the best mixture ratio for the optimization of the UCS and cost of concrete, this paper uses TOPSIS to rank the scores of twenty Pareto front optimal solutions. The MOBAS model proposed based on the weighted method can smoothly find the Pareto optimal solution for the UCS of concrete. The multi-objective optimization model proposed in this paper can effectively reduce the cost of concrete without changing the UCS of concrete and the multi-objective optimization model has a good effect and strong generalization ability. The Pareto front optimal solution with the highest score is 1 and the UCS of the corresponding concrete is 119.48 MPa and the cost is 37.29 USD/m^3^.

This study proposes a new intelligent design method for concrete design based on the consideration of economical and mechanical properties. However, in the current global environment of “Net Carbon Zero”, the design output index of carbon emissions has not been considered. Therefore, in future research, a multi-objective optimization design should be proposed, considering the objectives of economy-carbon emission-mechanical properties to be achieved.

## Figures and Tables

**Table 1:** Variables data analysis.

Variables	Min.	Max.	Median	Mode	Average	Std.	Variance
Cement (kg/m^3^)	133	540	313.30	313	311.65	199.20	14,207.73
Water (kg/m^3^)	126.60	214	176.50	178	174.43	18.05	325.63
Blast furnace slag (kg/m^3^)	0	282.80	150.10	0	117	38.50	1479.09
Coarse aggregate (kg/m^3^)	810	1134.30	944.70	852.10	946.50	81.24	6599.97
Fine aggregate (kg/m^3^)	605	992.60	788.95	755.80	744.03	75.64	5721.46
Superplasticizer (kg/m^3^)	2	32.20	8.23	8	9.13	4.99	24.87
Uniaxial compressive strength (MPa)	18.29	81.75	45.08	71.30	47.67	15.78	249.00

**Table 2:** Cost and density of each material.

The Name of the Material	Cost (USD/m^3^)	Density (kg/m^3^)
C	0.0475	3150
W	0.00024	1000
SF	0.0238	2750
CA	0.0048	2700
FA	0.006	2600
SP	1.667	1150

**Table 3:** Proportion relation of each component.

**Molecular**	**Denominator**	**Minimum**	**Maximum**
W	C+SF	0.154	1.609
SF	C+SF	0	2.126
SP	C+SF	0.002	0.242
CA	CA+FA	0.381	0.802
CA	C+SF	0.984	8.529

**Table 4:** Concrete mixtures by TOPSIS score.

No.	C (kg/m^3^)	W (kg/m^3^)	SF (kg/m^3^)	CA (kg/m^3^)	FA (kg/m^3^)	SP (kg/m^3^)	UCS (MPa)	Cost (USD/m^3^)	TOPSIS Score
1	484.21	191.44	239.70	1080.26	983.94	3.72	119.48	37.29	1.00
2	425.05	160.85	218.01	118.05	990.24	3.80	111.26	36.12	0.96
3	486.61	191.96	265.97	1109.02	977.62	12.68	128.61	48.90	0.89
4	479.67	207.51	249.74	1127.77	973.91	13.18	127.20	48.59	0.89
5	537.41	213.57	156.19	1095.44	990.81	10.48	119.62	46.36	0.89
6	382.38	184.80	279.26	951.99	893.64	3.58	101.42	36.02	0.88
7	426.78	165.80	111.73	1070.09	981.97	3.79	97.92	35.72	0.86
8	397.63	163.36	117.62	1132.20	939.25	4.05	94.65	35.20	0.84
9	306.81	200.93	212.77	1016.68	946.51	2.70	88.89	30.55	0.82
10	512.76	195.40	229.63	1120.27	953.36	23.32	131.55	63.19	0.77
11	233.33	199.56	222.49	1126.76	963.29	3.39	81.70	28.56	0.77
12	207.86	168.20	262.14	937.89	986.73	2.52	74.14	28.46	0.71
13	289.54	196.03	123.76	1102.29	928.70	2.20	69.16	28.16	0.67
14	220.52	202.97	186.89	1051.07	943.54	2.45	64.87	26.77	0.65
15	163.40	212.24	230.53	801.00	921.20	2.00	49.40	25.48	0.56
16	153.90	189.56	226.59	856.40	893.55	2.00	42.91	25.48	0.53
17	153.90	189.56	226.59	856.40	893.55	2.00	42.91	25.48	0.53
18	133.00	205.11	210.57	856.36	961.70	2.00	39.49	23.85	0.53
19	133.00	182.09	192.11	951.65	936.22	2.00	33.25	23.85	0.50
20	133.00	187.70	202.01	1097.01	775.29	2.00	25.00	23.78	0.48

## Data Availability

The data presented in this study are available on request from the corresponding author.
